# Amplification-free whole-genome shotgun bisulfite sequencing of mouse primordial germ cells

**DOI:** 10.1186/1756-8935-6-S1-P116

**Published:** 2013-04-08

**Authors:** Hisato Kobayashi, Takayuki Sakurai, Fumihito Miura, Misaki Imai, Kentaro Mochiduki, Eikichi Yanagisawa, Akihiko Sakashita, Takuya Wakai, Yutaka Suzuki, Takashi lto, Yasuhisa Matsui, Tomohiro Kono

**Affiliations:** 1 Department of BioScience, Tokyo University of Agriculture, Setagaya-ku, Tokyo, Japan; 2 Department of Biophysics and Biochemistry, Graduate School of Science, University of Tokyo, Bunkyo-Ku, Tokyo, Japan; 3 Genome Research Center, NODAI Research Institute, Tokyo University of Agriculture, Setagaya-ku, Tokyo, Japan; 4 Cell Resource Center for Biomedical Research, Institute of Development, Aging and Cancer, Tohoku University, Sendai, Miyagi, Japan; 5 Department of Medical Genome Sciences, Graduate School of Frontier Sciences, The University of Tokyo, Kashiwa, Chiba, Japan

## Background

Dynamic epigenetic reprogramming occurs during mammalian germ cell development, whereas the targets of this process including DNA demethylation and de novo methylation remain poorly understood. Here, we examined genome-wide methylation profiles in developing primordial germ cells (PGCs) of mice using high-throughput shotgun sequencing of bisulfite-treated DNA (whole-genome shotgun bisulfite sequencing; WGSBS), which accurately quantifies whole-genome methylation levels at single-base resolution.

## Materials and methods

Using Illumina sequencing libraries, we scaled down the construction and analysis to nanogram quantities of DNA by generating a new WGSBS library, termed the post-bisulfite adapter tagging (PBAT) method. PBAT libraries were generated from 2,000-5,000 PGCs and WGSBS analysis was performed using Illumina HiSeq 2000. Thus, we could provide complete maps of cytosine methylation in developing male and female PGCs during gonadal sex determination (at E10.5, E13.5, and E16.5).

## Results

This DNA methylome study demonstrated genome-wide DNA demethylation, with erasure of genomic imprinting and X-inactivation during gonadal sex determination and gender-specific differences in genome-wide and gene-specific (a part of CpG islands) DNA methylation levels in developing PGCs. Some of these global/local changes in DNA methylation during PGC progression were consistent with previous as well as more recent studies. However, our complete DNA methylome maps revealed important and novel details of DNA methylation and demethylation processes during PGC development. Some of the new findings from this study include the following: (i) PGC DNA methylomes exhibited sex- and chromosome-specific differences in genome-wide CpG and CpG island methylation during early to late PGC development; (ii) LINE_L1, LTR_MRVK, and LTR_ERV1 retrotransposons were resistant to DNA demethylation at high CpG densities during PGC migration; (iii) some maternally imprinted genes remained partially methylated in primary oocytes during fetal stages; and (iv) non-CpG methylation occurred in male gonocytes during mitotic arrest.

## Conclusions

In this study, we performed WGSBS mapping with thousands of mammalian cells (equal to approximately 20-50 ng genomic DNA) using the PBAT method. Our data and techniques can therefore serve as a platform for future studies to elucidate the role of epigenetic modifications in germline development and other biological processes.
